# Impact of COVID-19 Diagnosis on Weight Trajectories of Children in the US National COVID Cohort Collaborative

**DOI:** 10.1101/2025.01.03.25319927

**Published:** 2025-01-05

**Authors:** Md Mozaharul Mottalib, Thao-Ly T. Phan, Carolyn T Bramante, Christopher G Chute, Lee A Pyles, Rahmatollah Beheshti

**Affiliations:** University of Delaware, Newark, DE, US; Nemours Children’s Health, Wilmington, DE, US; University of Minnesota, Minneapolis, MN, US; Johns Hopkins School of Medicine, Baltimore, MD, US; West Virginia University, Morgantown, WV, US; University of Delaware, Newark, DE, US

**Keywords:** COVID-19, weight gain patterns, weight trajectories, childhood obesity, National COVID Cohort Collaborative

## Abstract

**Background:**

The COVID-19 pandemic has exacerbated the obesity epidemic, with both adults and children demonstrating rapid weight gain during the pandemic. However, the impact of having a COVID-19 diagnosis on this trend is not known.

**Methods:**

Using longitudinal data from January 2019 to June 2023 collected by the US National Institute for Health’s National COVID Cohort Collaborative (N3C), children (age 2–18 years) with positive COVID-19 test results (n=11,474, 53% male, mean [SD] age 5.57 [±3.29] years, 54% white, mean [SD] 5.2 [±2.9] BMI observations per participants) were matched with COVID-19 negative children with identical demographic characteristics and similar observation window. We compared BMI percentile trajectories between the COVID-19 positive and COVID-19 negative cohorts, with further evaluation performed on COVID-19 positive patients stratified by hospitalization status.

**Results:**

COVID-19 positive patients had a greater increase in $\%BMI_{p95}$ than COVID-19 negative patients (average increase of 2.34 (±7.73) compared to 1.46 (±6.09), p<0.0005). COVID-19 positive patients gained more weight after their diagnosis of COVID-19 than before. Non-hospitalized children gained more weight than hospitalized children (average increase in $\%BMI_{p95}$ of 2.38 (±7.65)) compared to 1.87 (±8.54)). Mixed effect regression analyses demonstrated that these associations remained even after adjusting for time, demographics, and baseline $\%BMI_{p95}$.

**Conclusions:**

Having a COVID-19 diagnosis was associated with more rapid weight gain, especially after diagnosis and early in the pandemic. Future research should explore the reasons for this association and the implications for future health emergencies.

## Introduction

The global impact of the severe acute respiratory syndrome coronavirus 2 (SARS-CoV-2), later designated as Coronavirus Disease 2019 (COVID-19), pandemic has been profound. To date, it has afflicted over 750 million individuals, resulting in a staggering toll of more than 7 million lives lost(1, 2). While its impact on adults has been extensively studied(3–5), there has been limited exploration of its effects on children and adolescents(6–8). However, the pandemic-induced shifts in lifestyle behaviors could have enduring effects on children’s weight(9). As a response to the pandemic, public safety measures such as lockdowns, quarantine, and social distancing recommendations disrupted the daily lives of families and children. These unique challenges forced families to adapt to new circumstances, prompting changes in their lifestyles and eating behaviors. Increased sedentary behavior, extended screen time, and reduced physical activity have been associated with a rise in obesity across populations(10–14). Not long after the start of the pandemic, the American Academy of Pediatrics (AAP) raised concerns that these changes may adversely impact children’s nutrition, physical activity, and obesity risk(15) and exacerbate an existing obesity epidemic that affects nearly 20% of children in the United States(16, 17) and poses substantial risks for comorbid conditions, including cardiometabolic diseases and mental health problems(18–21).

Studies have consistently demonstrated significant increases in weight among children and adults during the pandemic. For example, Lin et al.(3) conducted a longitudinal cohort study of adults during the initial shelter-in-place order in the US from March 19, 2020, to April 6, 2020. They found a significant increase in weight over a 3-month period at a rate of about 1.5 lbs. weight gain per month. Woolford et al.(22) compared the BMI of youth aged 5 to 17 years during the pandemic in 2020 with the BMI in the same period before the pandemic in 2019 and found significant increases in rates of overweight and obesity in 2020(4, 23–25). Since then, several pediatric studies have elucidated potential lifestyle, behavioral, and psychosocial causes of weight change among children due to the pandemic(26–29) and have confirmed significant increases in weight during the pandemic that exceeded the usual pre-pandemic weight change(6, 22, 30–32).

While it is clear that the pandemic has led to significant weight gain among children, studies have not examined the impact of having a COVID-19 diagnosis on this trend. Indeed, research related to COVID-19 diagnoses has been limited by the paucity of large, multi-institutional datasets of individuals testing positive for COVID-19(33–36). In this study, we leverage the extensive database of the National COVID Cohort Collaborative (N3C),(37) which includes patients from across the country who tested positive for COVID-19 and demographically matched controls. Our study presents an in-depth analysis of the changes in children’s weight during this unprecedented time, especially focusing on understanding how a COVID-19 infection and hospitalization for a COVID-19 infection impacted weight trajectories among children during different stages of the pandemic(38).

## Materials and Methods

### National COVID Cohort Collaborative (N3C)

National COVID Cohort Collaborative (N3C) is a multisite partnership that is comprised of members from the NIH Clinical and Translational Science Awards Program, it’s Center for Data to Health, the IDeA Centers for Translational Research, and several Electronic Health Record (EHR)-based research networks.(39) It aggregates and harmonizes electronic health record (EHR) data across clinical organizations and health system entities in the US to create a longitudinal multicenter cohort of patients with a COVID-19 diagnosis, matched in a 2:1 ratio to patients without a COVID-19 diagnosis by site, age, sex, and race. N3C has a national governance and regulatory body, uses COVID-19 cohort definitions via community-developed phenotypes, harmonizes data across 4 common data models (CDMs), and has a collaborative analytics platform to support the deployment of novel algorithms of data aggregated from the United States(37). The N3C has unique features that distinguish it from other COVID-19 data resources. It harmonizes data from many clinical sites across the US (98 signed data transfer agreements as of February 2024). This is important since many US reports of COVID-19 clinical characteristics come from a single hospital or healthcare system in a single geographic region. Furthermore, central curation ensures that N3C data are robust, and the quality is assured across sites. The N3C database contains data on weight, height, BMI, BMI percentile, and obesity diagnosis. These codes are adjusted and synchronized by the N3C regulatory team for easier analysis(40).

### Data Extraction and Cohort Definition

**Figure 1:**
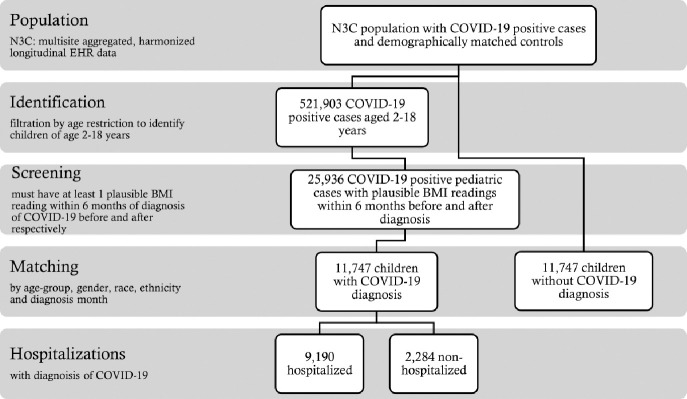
Flowchart of cohort selection.

We accessed the level 3 Limited Data Set (LDS) from the N3C data enclave, which includes patient data with dates of service and patient ZIP code. This data is only available to US-based research institutes with approved Data Use Request (DUR). We performed a retrospective analysis of children undergoing COVID-19 testing at an N3C site between January 1, 2019 (start date of N3C database) and June 30, 2023 (date of data extraction), whose data were completely harmonized, integrated, and released for analysis. Patients were included as COVID-19 positive cases if they met the following criteria: 1) aged 2 to 18 years old, 2) tested positive for COVID-19 following the N3C phenotyping guidelines to define COVID-19 positivity based on a COVID-19–positive polymerase chain reaction or antigen test, or an International Classification of Diseases (ICD)-10-CM diagnostic code for COVID-19 during the same single index encounter(41), and 3) had at least two BMI measurements or body height/weight measurements, with at least one measurement within 6 months before diagnosis and one measurement within 6 months after diagnosis. From an initial population of 521,903 patients (adult and pediatric), 25,936 patients were found to meet the criteria (Figure 1).

We then matched the cohort with patients without a diagnosis of COVID-19 in the repository (controls, COVID-19 negative patients), based on age group, gender, race, ethnicity, and observation window. For example, a positive-diagnosis patient with observations from November 2019 to November 2020 was matched with a negative-diagnosis patient having observations starting from November 2019, spreading over a 13-month observation window. This reduced each cohort to 11,474, as not every matched control had two or more weights and heights in the database within a 13-month timeframe. Matching was done using the R package MatchIt(42) with a generalized linear model for calculating the propensity score among the samples. Matching was performed with a greedy nearest neighbor matching algorithm implemented in the MatchIt package. The choice of this algorithm yielded the maximum number of matches, keeping the cohort considerably large. In the initial population, only around 32% of the patients had more than 2 measurements. However, in the final matched cohort, there were around 46% of the patients who had more than 2 measurements.

### Demographic Characteristics

For the cohort defined above, demographic characteristics were extracted, including date of birth (to derive age), sex, race, and ethnicity. For the purposes of analysis, age, sex, race, and ethnicity were grouped into broad categories (Table 1). It is important to note that the COVID-19 negative (control) group exhibited identical demographic characteristics and had weight measurements available at comparable time points for each corresponding patient.

### Weight Status Metrics

For the cohort defined above, we extracted height and weight measurements starting from 12 months before the COVID-19 diagnosis up until the date of data extraction. Weights were restricted between 5kg and 300kg and heights between 0.6m to 2.43m according to CDC growth parameters established for the defined age range(43). BMI was calculated using, $BMI=weight/{height}^{2}$ formula as $kilogram/{meter}^{2}$. Extreme values of BMI were excluded based on their modified z-scores(44).

In the U.S., the CDC 2000 BMI charts are recommended for use in children 2 to 20 years of age to determine a child’s BMI percentile in relation to norms for age and sex. Freedman et al.(45) illustrated the challenges in using BMI percentiles in children of different ages and compared alternative metrics to quantify adiposity status for children with a BMI at the upper extremes. Because of these challenges, many in the research and clinical community use percent above the 95^th^ percentile for those with extreme BMI, with 120% of the 95th percentile of BMI ($\%BMI_{p95}$) is used to define severe obesity in children (46). The percent of the BMI 95^th^ percentile is defined as:

$$\%BMI_{p95}=\frac{BMI}{BMI_{p95}}\times 100\%$$

where, $BMI_{p95}$ shows the BMI 95^th^ percentile. Because of the common limitations of EHR data (i.e., missingness and irregular sampling), $BMI_{p95}$ values were needed to be imputed to derive trajectories. Only 2 measurements would represent a change score instead of a trajectory. Polynomial interpolation of order 2 was used to impute the missing values, where at least 4 measurements were present (~40% of the cohort) in the trajectory; for the remainder of cases, linear imputation was used. In total around 10,500 patient trajectories (~46% of the cohort) were imputed using the aforementioned techniques.

### Statistical Methodologies

Descriptive statistics were summarized by the mean and standard deviation (SD) for continuous variables and frequency with percent for categorical variables. One-way ANOVA was used for comparing continuous variables, and the chi-squared test was used for categorical variables, implemented using the Python Statsmodels(47) and SciPy(48) libraries. We generated figures depicting the weight trajectories of the COVID-19 positive and COVID-19 negative cohorts, stratified by demographic groups. We performed several statistical analyses on the mean $\%BMI_{p95}$ changes during the Early Pandemic (January 2020 – December 2020) and Late Pandemic (January 2021 – June 2023). Bartlett testing was performed to evaluate the homogeneity of variance among the mean $\%BMI_{p95}$ change(49). Since the variances were not equal among groups based on stages of the pandemic, Kruskal-Wallis H-test(50) was performed on the change in $\%BMI_{p95}$. We used mixed-effects models to examine the rates of change in the $\%BMI_{p95}$*,* to examine the impact of having a COVID-19 infection on change in $\%BMI_{p95}$. Additionally, a similar but separate examination of weight change in the COVID-19 positive patients was performed to examine the impact of having a hospitalization on change in $\%BMI_{p95}$. For both models, we included time, baseline $\%BMI_{p95}$ and demographics as covariates. The time of COVID-19 diagnosis, especially the pandemic era indicator, was an important variable in the analyses where we investigated the weight change trend during different pandemic eras. Demographics were included as fixed effects in the models.

## Results

### Comparison of BMI Percentile Trajectories between COVID-19 Positive and COVID-19 Negative Cohorts

**Figure 2:**
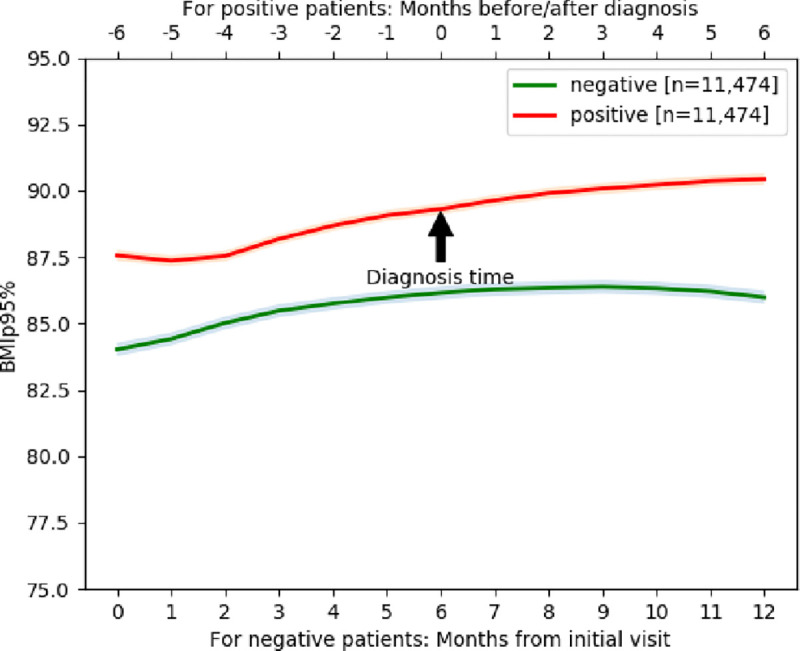
$\%BMI_{p95}$ trajectory with 95% confidence interval band over 13-month period including 6-months prior to diagnosis and 6-month after diagnosis for COVID-19 positive cases and over the same 13-month period for matched COVID-19 negative patients.

**Figure 3:**
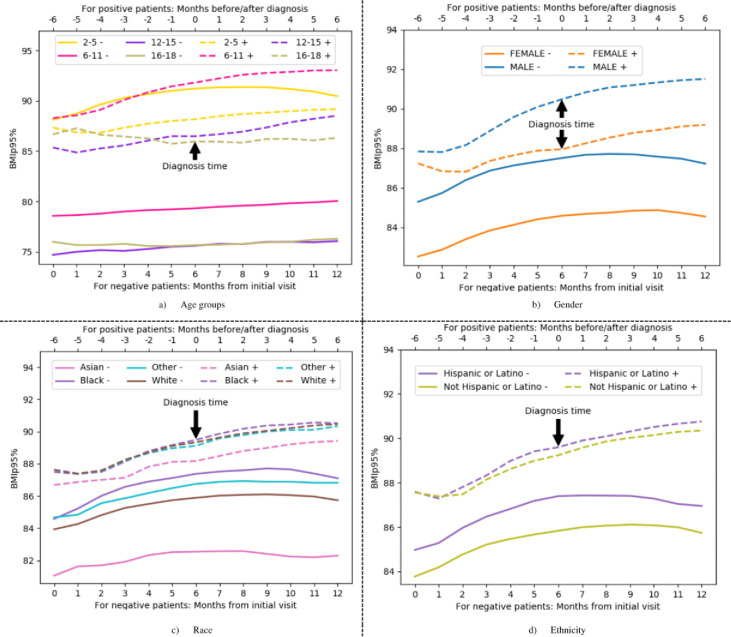
$\%BMI_{p95}$ trajectories based on demographic group. Each line represents the mean value at that time period.

$\%BMI_{p95}$ trajectories of COVID-19 positive cases and COVID-19 negative controls are illustrated in Figure 2, with stratification by demographic groups in Figure 3. Baseline $\%BMI_{p95}$ was higher in the COVID-19 positive group (mean 88.07, SD 7.25) compared to the COVID-19 negative group (mean 85.11, SD (10.31, p < 0.005) except among the youngest children (aged 2–5 years). Although the actual difference was 2.96 percentile points, even modest increases in BMI percentile among children, particularly those already in higher percentiles, are clinically meaningful in the context of obesity and associated health risks, such as more severe COVID-19 outcomes. Both groups demonstrated an increase in $\%BMI_{p95}$ over the first 6 months of the time period, but only COVID-19 positive patients continued to have an increase in $\%BMI_{p95}$ in the latter 6 months, after diagnosis. In fact, COVID-19 positive patients gained more weight after their diagnosis of COVID-19 than before the diagnosis (average increase in weight of 2.34 (±7.73) after diagnosis vs. 1.46 (±6.09) before diagnosis, p < 0.005). Children between the ages of 6 and 11 demonstrated more rapid increases in $\%BMI_{p95}$ than other age groups, especially among the COVID-19 positive cohort. Male patients in the COVID-19 positive cohort demonstrated more rapid increases in $\%BMI_{p95}$ than female patients. There were no major differences in BMI percentile trajectories by race or ethnicity. Mixed effects regression analyses showed that the association between having a COVID-19 diagnosis and having a more rapid increase in $\%BMI_{p95}$ remained, even when accounting for demographics, time, and baseline $\%BMI_{p95}$ (coefficient = 31.05, p= 0.005). In the regression model, only age group and gender also had a significant association with change in $\%BMI_{p95}$.

### Comparison of Change in BMI Percentile between COVID-19 Positive and COVID-19 Negative Cohorts Based on Pandemic Stage

Table 1 presents the average change in $\%BMI_{p95}$ for the COVID-19 positive and negative cohorts by stages of the pandemic. Both cohorts had an increase in $\%BMI_{p95}$ during the early and late pandemic stages, but patients in the COVID-19 positive cohort had greater increases in both early (average $\%BMI_{p95}$ increase of 2.44 percentiles) and late pandemic (average $\%BMI_{p95}$ increase of 2.21 percentiles) stages compared to the COVID-19 negative cohort (average $\%BMI_{p95}$ increase of 1.23 and 1.87, respectively). The COVID-positive cohort gained more weight during the early pandemic compared to the late pandemic, whereas the COVID-negative cohort gained more weight during the late pandemic compared to the early pandemic. These trends were the same for patients regardless of demographic group, except for Black patients without a COVID-19 diagnosis and female patients with a COVID-19 diagnosis.

### Comparison of Change in BMI Percentile between COVID-19 Positive Patients Based on Hospitalization

**Figure 4:**
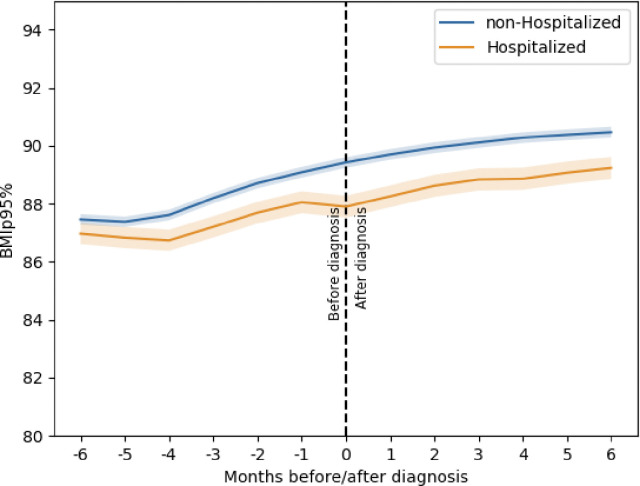
$\%BMI_{p95}$ trajectories based on hospitalization. Each line representing the trajectory of the mean values at specific time periods, the shaded region represents the 95% confidence interval.

Approximately 19% of children among the COVID-19 positive cohort were hospitalized. As shown in Figure 4, non-hospitalized children had greater changes in BMI percentile over time (average $\%BMI_{p95}$ change of 2.38 (±7.65)) compared to hospitalized children (average increase of 1.87 (±8.54) $\%BMI_{p95}$). Hospitalized children were more likely to be younger and Black (Table 2). Mixed effects regression analyses showed that the association between being hospitalized for COVID-19 and less rapid increase in $\%BMI_{p95}$ remained, even when accounting for demographics, time, and baseline $\%BMI_{p95}$ (coefficient = 29.684, 95% p = 0.003). In the regression model, only age group and gender also had a significant association with change in $\%BMI_{p95}$.

## Discussion

While much has been written about the impact of the COVID-19 pandemic on weight gain among children in the United States, this is the first study that we are aware of that highlights how having a diagnosis of COVID-19 contributed to this trend. Using a large nationally representative database, we found that children with a diagnosis of COVID-19 had larger and more persistent increases in BMI percentile over time compared to children without a COVID-19 diagnosis, especially early in the pandemic. Children with a diagnosis of COVID-19 gained more weight after diagnosis than before diagnosis. We also found that among children with a COVID-19 diagnosis, those who were hospitalized had less rapid weight gain than those who were not hospitalized. These findings add to our understanding of the weight gain experienced by children during the pandemic and highlight the complexity of factors that likely contributed.

Our study leverages the National COVID Cohort Collaborative (N3C), which includes nearly 100 institutions working together to build a large, centralized data resource to study COVID-19. Under the stewardship of the NIH’s National Center for Advancing Translational Science, N3C has harmonized EHR data across health systems and uniquely consists of cases (patients with a COVID-19 diagnosis) and demographically matched controls without a COVID-19 diagnosis. Because of this, we can uniquely comment on a nationally representative sample and on the impact of a COVID-19 diagnosis and hospitalization for COVID-19 on weight outcomes. To our knowledge, there exist only a few studies in adults that have examined this question and no studies in pediatric populations.

As noted in the Introduction, the COVID-19 pandemic contributed to weight gain in children in several ways., including increased psychosocial stressors like social isolation and food insecurity, impaired sleep routines, and the promotion of sedentary behaviors(13). In addition to these universally experienced factors, children with a COVID-19 diagnosis may have experienced increased isolation and exposure to traumatic aspects of the pandemic (e.g., having family members pass away from COVID-19), especially early in the pandemic. In addition, for some children, symptoms like fatigue and headaches from a COVID-19 infection may have contributed further to sedentary behaviors. Finally, there is evidence of an increased incidence of Diabetes (both Type 1 and Type 2) with COVID-19 infection because of the pro-inflammatory nature of COVID-19 and its effects on the pancreas(51). While not directly tied to weight gain, it is plausible that these biological factors could have an adverse effect on weight.

Interestingly, among children diagnosed with COVID-19, we found less rapid weight gain in those who were hospitalized compared to those who were not hospitalized. This is consistent with studies in adults demonstrating unintentional weight loss among those hospitalized for COVID-19(52), which may be attributed to inadequate nutritional intake or immobilization during hospitalization. In addition, increased inflammatory cytokines due to severe COVID-19 infection may lead to cachexia and loss of lean body mass(53), as shown in adults. Finally, children hospitalized for COVID-19 comprised only 19% of the total cohort of children diagnosed with COVID-19 in our sample and likely represent more vulnerable populations with more risk for severe disease, whether because of younger age or more medical complexity. Because of their vulnerability, these children may have had closer medical monitoring(54) – especially after hospitalization - that mitigated excessive weight gain.

While the N3C database and our approach to studying the impact of COVID-19 on BMI percentile trajectories in children has several strengths, this study also has limitations to consider. As data are aggregated from many health systems using different CDMs that vary in granularity, some sites had systematic missingness of certain variables. Indeed, our overall sample size decreased from 25,936 children to 11,474 cases due to missing data, especially since we required weight and heights for at least two time-points within a certain timeline compared to diagnosis for both cases and controls to conduct the longitudinal analysis. Furthermore, due to missing data, we used interpolation to fill the gap in the trajectories of $\%BMI_{p95}$ for some patients, which might not represent the actual data. While we adjusted for baseline BMI, the difference in BMI percentiles may still hold clinical significance due to the increased baseline risk of severe outcomes associated with obesity in COVID-19 cases. Given that asymptomatic cases may exhibit distinct weight trajectories compared to symptomatic cases, we opted not to account for asymptomatic cases. This introduces an added layer of complexity to the interpretation of our findings.

We acknowledge that BMI can fluctuate in children over time and that the time window for BMI measurement following diagnosis ranges from one week to six months. This variability could influence the interpretation of the weight trajectories observed, particularly in those with shorter follow-up periods. As such, the generalizability of the findings should be interpreted with caution, especially for participants with only two BMI measurements or shorter time intervals between measurements. Our study did not account for comorbidities associated with sedentary behavior and weight gain, such as diabetes and hypertension, which can influence BMI trajectories and the effect of COVID-19 on weight gain. Future studies should consider these comorbidities to better understand the impact of COVID-19 on weight gain in pediatric populations. These limitations, particularly the lack of adjustment for comorbidities and the focus on U.S.-based data, suggest that caution should be taken when generalizing these results to other populations. Further research is needed to explore the impact of COVID-19 on weight gain in children with different health profiles, particularly in international cohorts and populations not well-represented in the N3C database.

## Conclusion

This study reports on the BMI percentile trajectory of a large nationally representative cohort of children in the United States using the N3C database. Our study uniquely adds to the literature by evaluating how a diagnosis of COVID-19 was associated with BMI percentile trajectories during the pandemic. Children diagnosed with COVID-19 had larger and more persistent increases in BMI percentile over time compared to children without a COVID-19 diagnosis, highlighting the additional impact that a diagnosis of COVID-19 had on weight gain in children beyond the impact of the pandemic generally alone. Future studies should examine the reasons for this association and whether this finding is specific to COVID-19 or could have implications for future health conditions and emergencies.

## Figures and Tables

**Table 1:** Average change in %*BMI*_*p*95_ before and after the COVID-19 pandemic periods in patients both with and without COVID-19 diagnosis aged 2–18 years with measurements available for about a year.

Characteristics	Average change in %BMI_p95_ (±SD)					
	Control			Covid positive		
	Early pandemic	Late pandemic	Difference	Early pandemic	Late pandemic	Difference
**Overall**	1.23 (±0.15)	1.87 (±0.27)	0.64*	2.44 (±0.29)	2.21 (±0.2)	−0.23*
**Age group**						
2–5 (60.14%)	1.45 (±0.23)	2.26 (±0.40)	−0.81*	1.82 (±0.39)	1.69 (±0.26)	−0.13*
6–11 (33.29%)	0.96 (±0.18)	1.23 (±0.33)	−0.27	3.44 (±0.49)	3.32 (±0.34)	−0.12*
12–15 (4.75%)	1.01 (±0.52)	1.30 (±1.28)	−0.29	3.08 (±1.00)	2.06 (±1.08)	−1.02*
16–18 (1.82%)	−0.13 (±1.10)	1.52 (±1.93)	1.65	0.945 (±2.50)	−0.64 (±1.83)	−1.58
**Gender**						
Male (53.28%)	1.27 (±0.21)	1.63 (±0.36)	0.36*	3.19 (±0.40)	2.65 (±0.27)	−0.57*
Female (46.71%)	1.19 (±0.22)	2.14 (±0.42)	0.95*	1.56 (±0.42)	1.73 (±0.29)	0.17*
**Race**						
White (53.41%)	1.11 (±0.18)	1.88 (±0.33)	0.77*	2.72 (±0.56)	2.09 (±0.43)	−0.02*
Black or African American (17.93%)	1.82 (±0.37)	1.78 (±0.57)	−0.03*	3.19 (±0.62)	2.37 (±0.45)	−0.82*
Asian (3.33%)	0.70 (±0.65)	0.89 (±1.60)	0.19	1.99 (±1.61)	1.88 (±1.07)	−0.12
Native Hawaiian or Other Pacific Islander (0.1%)	2.33 (±4.28)	−0.29 (±4.05)	−4.35	1.49 (±5.29)	1.99 (±2.53)	0.49
Other (25.23%)	0.94 (±0.53)	2.48 (±1.48)	1.54	2.36 (±0.95)	1.76 (±0.68)	−0.6
**Ethnicity**						
Hispanic or Latino (28.66%)	1.27 (±0.32)	1.86 (±0.59)	0.59*	2.72 (±0.56)	2.09 (±0.43)	−0.63*
Non-Hispanic or Latino (66.21%)	1.22 (±0.17)	1.87 (±0.31)	0.65*	2.33 (±0.34)	2.24 (±0.23)	−0.09*

[Early pandemic: January 2020 – December 2020, Late pandemic: January 2021 – June 2023, (*) represents statistically significant difference with a p-value < 0.005]

**Table 2:** Demographic characteristics of cohort based on hospitalization status

Characteristics	Patients, no. (%)		Hospitalization Risk Ratio	p-value
	Not hospitalized (n = 9190)	Hospitalized (n = 2284)		
**Age**				< 0.005
Age (y), mean (SD)	5.63 (3.287)	5.24 (3.22)	-	
2–5	5433 (59.12%)	1443 (63.18%)	Ref.	
6–11	3134 (34.1%)	705 (30.87%)	0.88	
12–15	455 (4.95%)	111 (4.86%)	0.93	
16–18	168 (1.83%)	25 (1.09%)	0.62	
**Gender**				0.0402
Male	4843 (52.7%)	1270 (55.6%)	Ref.	
Female	4346 (47.29%)	1014 (44.4%)	0.91	
**Race**				< 0.005
White	5004 (54.45%)	1125 (49.26%)	Ref.	
Black or African American	1567 (17.05%)	491 (21.5%)	1.30	
Asian	286 (3.11%)	59 (2.58%)	0.93	
Other	666 (7.25%)	174 (7.62%)	1.13	
**Ethnicity**				0.0207
Hispanic or Latino	2607 (28.37%)	702 (30.74%)	Ref.	
Non-Hispanic or Latino	6109 (66.47%)	1487 (65.11%)	0.92	

## Data Availability

The data that supports the findings of this study is accessible through the N3C data repository access portal with necessary permissions.

## Impact Statement

This study investigating the weight trajectories of children during the COVID-19 pandemic shows that the pandemic has led to a significant increase in weight gain among US children aged 6–11, with boys gaining more weight than girls. Non-hospitalized COVID-19-positive children were found to be at greater risk of gaining weight.

## References

[1] Programme WHE. Number of COVID-19 cases reported to WHO: World Health Organization; 2024 [updated 28 January, 2024. Available from: https://data.who.int/dashboards/covid19/cases?n=c.

[2] Programme WHE. Number of COVID-19 deaths reported to WHO: World Health Organization; 2024 [updated 28 January, 2024. Available from: https://data.who.int/dashboards/covid19/deaths?m49=840&n=c.

[3] Lin AL, Vittinghoff E, Olgin JE, Pletcher MJ, Marcus GM. Body Weight Changes During Pandemic-Related Shelter-in-Place in a Longitudinal Cohort Study. JAMA network open. 2021;4(3):e212536–e. 33749764 10.1001/jamanetworkopen.2021.2536 PMC7985720

[4] Almandoz JP, Xie L, Schellinger JN, Mathew MS, Gazda C, Ofori A, Impact of COVID-19 stay-at-home orders on weight-related behaviours among patients with obesity. Clinical obesity. 2020;10(5):e12386. 32515555 10.1111/cob.12386 PMC7300461

[5] Aghili SMM, Ebrahimpur M, Arjmand B, Shadman Z, Sani MP, Qorbani M, Obesity in COVID-19 era, implications for mechanisms, comorbidities, and prognosis: a review and meta-analysis. International Journal of Obesity. 2021;45(5):998–1016. 33637951 10.1038/s41366-021-00776-8 PMC7909378

[6] Lange SJ, Kompaniyets L, Freedman DS, Kraus EM, Porter R, Blanck HM, Longitudinal trends in body mass index before and during the COVID-19 pandemic among persons aged 2–19 years—United States, 2018–2020. Morbidity and Mortality Weekly Report. 2021;70(37):1278. 34529635 10.15585/mmwr.mm7037a3 PMC8445379

[7] Umano GR, Rondinelli G, Rivetti G, Klain A, Aiello F, Miraglia del Giudice M, Effect of COVID-19 lockdown on children’s eating behaviours: A longitudinal study. Children. 2022;9(7):1078. 35884062 10.3390/children9071078 PMC9323163

[8] Anderson LN, Yoshida-Montezuma Y, Dewart N, Jalil E, Khattar J, De Rubeis V, Obesity and weight change during the COVID-19 pandemic in children and adults: A systematic review and meta-analysis. Obesity Reviews. 2023:e13550. 36721999 10.1111/obr.13550

[9] Rundle AG, Park Y, Herbstman JB, Kinsey EW, Wang YC. COVID-19 related school closings and risk of weight gain among children. Obesity (Silver Spring, Md). 2020;28(6):1008. 32227671 10.1002/oby.22813 PMC7440663

[10] Weaver RG, Armstrong B, Hunt E, Beets MW, Brazendale K, Dugger R, The impact of summer vacation on children’s obesogenic behaviors and body mass index: A natural experiment. International Journal of Behavioral Nutrition and Physical Activity. 2020;17(1):1–14. 33243252 10.1186/s12966-020-01052-0 PMC7690133

[11] Tanskey LA, Goldberg J, Chui K, Must A, Sacheck J. The state of the summer: a review of child summer weight gain and efforts to prevent it. Current obesity reports. 2018;7(2):112–21. 29644576 10.1007/s13679-018-0305-z PMC6857933

[12] Wang YC, Vine S, Hsiao A, Rundle A, Goldsmith J. Weight-related behaviors when children are in school versus on summer breaks: does income matter? Journal of School Health. 2015;85(7):458–66. 26032276 10.1111/josh.12274

[13] Stavridou A, Kapsali E, Panagouli E, Thirios A, Polychronis K, Bacopoulou F, Obesity in Children and Adolescents during COVID-19 Pandemic. Children. 2021;8(2):135. 33673078 10.3390/children8020135 PMC7918914

[14] Gupta M, Phan T-LT, Lê-Scherban F, Eckrich D, Bunnell HT, Beheshti R. Associations of Longitudinal BMI-Percentile Classification Patterns in Early Childhood with Neighborhood-Level Social Determinants of Health. Childhood obesity. 2024. 10.1089/chi.2023.0157 PMC11807911 39187268

[15] Pediatrics AAo. Supporting healthy nutrition and physical activity during the COVID-19 pandemic. American Academy of Pediatrics. 2020.

[16] Lobstein T, Brinsden H. Obesity: missing the 2025 global targets. World Obesity Federation; 2020.

[17] Hales CM, Carroll MD, Fryar CD, Ogden CL. Prevalence of obesity among adults and youth: United States, 2015–2016. 2017. 29155689

[18] Chang T-H, Chen Y-C, Chen W-Y, Chen C-Y, Hsu W-Y, Chou Y, Weight gain associated with COVID-19 lockdown in children and adolescents: A systematic review and meta-analysis. Nutrients. 2021;13(10):3668. 34684669 10.3390/nu13103668 PMC8540321

[19] Koletzko B, Holzapfel C, Schneider U, Hauner H. Lifestyle and body weight consequences of the COVID-19 pandemic in children: Increasing disparity. Annals of nutrition & metabolism. 2021:1. 10.1159/000514186 PMC7900479 33498055

[20] Al Hourani H, Alkhatib B, Abdullah M. Impact of COVID-19 lockdown on body weight, eating habits, and physical activity of Jordanian children and adolescents. Disaster medicine and public health preparedness. 2021:1–9. 10.1017/dmp.2021.48 PMC8129676 33588981

[21] Alves JM, Yunker AG, DeFendis A, Xiang AH, Page KA. BMI status and associations between affect, physical activity and anxiety among US children during COVID-19. Pediatric obesity. 2021;16(9):e12786. 33720550 10.1111/ijpo.12786 PMC8250275

[22] Woolford SJ, Sidell M, Li X, Else V, Young DR, Resnicow K, Changes in body mass index among children and adolescents during the COVID-19 pandemic. jama. 2021;326(14):1434–6. 34448817 10.1001/jama.2021.15036 PMC8511973

[23] Fayyaz H, Phan T-LT, Bunnell HT, Beheshti R. Who will Leave a Pediatric Weight Management Program and When?--A machine learning approach for predicting attrition patterns. arXiv preprint arXiv:220201765. 2022. PMC9854275 36686987

[24] Mottalib MM, Jones-Smith JC, Sheridan B, Beheshti R. Subtyping patients with chronic disease using longitudinal BMI patterns. IEEE Journal of Biomedical and Health Informatics. 2023;27(4):2083–93. 10.1109/JBHI.2023.3237753 PMC10350469 37021857

[25] Hargrove TW. BMI trajectories in adulthood: The intersection of skin color, gender, and age among African Americans. Journal of health and social behavior. 2018;59(4):501–19. 30303024 10.1177/0022146518802439 PMC6657514

[26] Cuschieri S, Grech S. COVID-19: a one-way ticket to a global childhood obesity crisis? Journal of diabetes & metabolic disorders. 2020;19:2027–30. 33173756 10.1007/s40200-020-00682-2 PMC7644278

[27] Hu P, Samuels S, Maciejewski KR, Li F, Aloe C, Van Name M, Changes in weight-related health behaviors and social determinants of health among youth with overweight/obesity during the COVID-19 pandemic. Childhood Obesity. 2022;18(6):369–82. 34919458 10.1089/chi.2021.0196 PMC9492789

[28] Storz MA. The COVID-19 pandemic: an unprecedented tragedy in the battle against childhood obesity. Clinical and experimental pediatrics. 2020;63(12):477. 33152743 10.3345/cep.2020.01081 PMC7738769

[29] Jenssen BP, Kelly MK, Powell M, Bouchelle Z, Mayne SL, Fiks AG. COVID-19 and changes in child obesity. Pediatrics. 2021;147(5). 10.1542/peds.2021-050123 33653879

[30] Brooks CG, Spencer JR, Sprafka JM, Roehl KA, Ma J, Londhe AA, Pediatric BMI changes during COVID-19 pandemic: An electronic health record-based retrospective cohort study. EClinicalMedicine. 2021;38. 10.1016/j.eclinm.2021.101026 PMC8318998 34337366

[31] Vogel M, Geserick M, Gausche R, Beger C, Poulain T, Meigen C, Age-and weight group-specific weight gain patterns in children and adolescents during the 15 years before and during the COVID-19 pandemic. International journal of obesity. 2022;46(1):144–52. 34556774 10.1038/s41366-021-00968-2 PMC8458556

[32] Weaver RG, Hunt ET, Armstrong B, Beets MW, Brazendale K, Turner-McGrievy G, COVID-19 leads to accelerated increases in children’s BMI z-score gain: an interrupted time-series study. American journal of preventive medicine. 2021;61(4):e161–e9. 34148734 10.1016/j.amepre.2021.04.007 PMC8443301

[33] Kompaniyets L, Agathis NT, Nelson JM, Preston LE, Ko JY, Belay B, Underlying medical conditions associated with severe COVID-19 illness among children. JAMA network open. 2021;4(6):e2111182–e. 34097050 10.1001/jamanetworkopen.2021.11182 PMC8185607

[34] Belay ED, Abrams J, Oster ME, Giovanni J, Pierce T, Meng L, Trends in geographic and temporal distribution of US children with multisystem inflammatory syndrome during the COVID-19 pandemic. JAMA pediatrics. 2021. 10.1001/jamapediatrics.2021.0630 PMC8025123 33821923

[35] Bailey LC, Razzaghi H, Burrows EK, Bunnell HT, Camacho PE, Christakis DA, Assessment of 135 794 pediatric patients tested for severe acute respiratory syndrome coronavirus 2 across the United States. JAMA pediatrics. 2021;175(2):176–84. 33226415 10.1001/jamapediatrics.2020.5052 PMC7684518

[36] Götzinger F, Santiago-García B, Noguera-Julián A, Lanaspa M, Lancella L, Carducci FIC, COVID-19 in children and adolescents in Europe: a multinational, multicentre cohort study. The Lancet Child & Adolescent Health. 2020;4(9):653–61. 32593339 10.1016/S2352-4642(20)30177-2 PMC7316447

[37] Haendel MA, Chute CG, Bennett TD, Eichmann DA, Guinney J, Kibbe WA, The National COVID Cohort Collaborative (N3C): rationale, design, infrastructure, and deployment. Journal of the American Medical Informatics Association. 2021;28(3):427–43. 32805036 10.1093/jamia/ocaa196 PMC7454687

[38] Estrada E, Ferrer E, Pardo A. Statistics for evaluating pre-post change: Relation between change in the distribution center and change in the individual scores. Frontiers in psychology. 2019;9:2696. 30671008 10.3389/fpsyg.2018.02696 PMC6331475

[39] Bennett TD, Moffitt RA, Hajagos JG, Amor B, Anand A, Bissell MM, Clinical characterization and prediction of clinical severity of SARS-CoV-2 infection among US adults using data from the US National COVID Cohort Collaborative. JAMA network open. 2021;4(7):e2116901–e. 34255046 10.1001/jamanetworkopen.2021.16901 PMC8278272

[40] Sharafeldin N, Bates B, Song Q, Madhira V, Yan Y, Dong S, Outcomes of COVID-19 in patients with cancer: report from the National COVID Cohort Collaborative (N3C). Journal of Clinical Oncology. 2021;39(20):2232. 34085538 10.1200/JCO.21.01074 PMC8260918

[41] Martin B, DeWitt PE, Russell S, Anand A, Bradwell KR, Bremer C, Children with SARS-CoV-2 in the National COVID Cohort Collaborative (N3C). Medrxiv. 2021.

[42] Ho D, Imai K, King G, Stuart EA. MatchIt: Nonparametric Preprocessing for Parametric Causal Inference. Journal of Statistical Software. 2011;42(8):1–28.

[43] Kuczmarski RJ. 2000 CDC Growth Charts for the United States: methods and development: Department of Health and Human Services, Centers for Disease Control and …; 2002. 12043359

[44] A SAS Program for the 2000 CDC Growth Charts (ages 0 to <20 years): Centers for Disease Control and Prevention; [Available from: https://www.cdc.gov/nccdphp/dnpao/growthcharts/resources/sas.htm.

[45] Freedman DS, Davies AJG, Kompaniyets L, Lange SJ, Goodman AB, Phan T-LT, A Longitudinal Comparison of Alternatives to Body Mass Index Z-Scores for Children with Very High Body Mass Indexes. The Journal of Pediatrics. 2021. 10.1016/j.jpeds.2021.02.072 33676932

[46] Freedman DS, Butte NF, Taveras EM, Goodman AB, Ogden CL, Blanck HM. The limitations of transforming very high body mass indexes into z-scores among 8.7 million 2-to 4-year-old children. The Journal of pediatrics. 2017;188:50–6. e1. 28433203 10.1016/j.jpeds.2017.03.039 PMC5572545

[47] Seabold S, Perktold J, editors. Statsmodels: Econometric and statistical modeling with python. Proceedings of the 9th Python in Science Conference; 2010: Austin, TX.

[48] Virtanen P, Gommers R, Oliphant TE, Haberland M, Reddy T, Cournapeau D, SciPy 1.0: fundamental algorithms for scientific computing in Python. Nature methods. 2020;17(3):261–72. 32015543 10.1038/s41592-019-0686-2 PMC7056644

[49] Tobias S, Carlson JE. Brief report: Bartlett’s test of sphericity and chance findings in factor analysis. Multivariate behavioral research. 1969;4(3):375–7. 26745847 10.1207/s15327906mbr0403_8

[50] Kruskal WH, Wallis WA. Use of ranks in one-criterion variance analysis. Journal of the American statistical Association. 1952;47(260):583–621.

[51] Zhang T, Mei Q, Zhang Z, Walline JH, Liu Y, Zhu H, Risk for newly diagnosed diabetes after COVID-19: a systematic review and meta-analysis. BMC medicine. 2022;20(1):444. 36380329 10.1186/s12916-022-02656-y PMC9666960

[52] Di Filippo L, De Lorenzo R, Cinel E, Falbo E, Ferrante M, Cilla M, Weight trajectories and abdominal adiposity in COVID-19 survivors with overweight/obesity. International Journal of Obesity. 2021;45(9):1986–94. 34002039 10.1038/s41366-021-00861-y PMC8127478

[53] Di Filippo L, De Lorenzo R, D’Amico M, Sofia V, Roveri L, Mele R, COVID-19 is associated with clinically significant weight loss and risk of malnutrition, independent of hospitalisation: a post-hoc analysis of a prospective cohort study. Clinical Nutrition. 2021;40(4):2420–6. 33160700 10.1016/j.clnu.2020.10.043 PMC7598735

[54] Woodruff RC, Campbell AP, Taylor CA, Chai SJ, Kawasaki B, Meek J, Risk factors for severe COVID-19 in children. Pediatrics. 2022;149(1):e2021053418. 34935038 10.1542/peds.2021-053418 PMC9213563

